# Limited Benefit of Sleep Extension on Cognitive Deficits During Total Sleep Deprivation: Illustration With Two Executive Processes

**DOI:** 10.3389/fnins.2019.00591

**Published:** 2019-06-19

**Authors:** Arnaud Rabat, Pierrick J. Arnal, Hortense Monnard, Mégane Erblang, Pascal Van Beers, Clément Bougard, Catherine Drogou, Mathias Guillard, Fabien Sauvet, Damien Leger, Danielle Gomez-Merino, Mounir Chennaoui

**Affiliations:** ^1^Unité Fatigue et Vigilance, Institut de Recherche Biomédicale des Armées, Brétigny-sur-Orge, France; ^2^VIFASOM Team (EA 7330), Hotel Dieu Hospital, Paris Descartes University, Paris, France; ^3^Alertness and Sleep Center, Hotel Dieu Hospital, Paris Descartes University, Paris, France

**Keywords:** sleep extension, total sleep deprivation, executive processes, inhibition, working memory, healthy subjects

## Abstract

**Introduction:** Sleep extension has been associated with better alertness and sustained attention capacities before, during and after sleep loss. However, less is known about such beneficial effect on executive functions (EFs). Our aim was to investigate such effects on two EFs (i.e., inhibition and working memory) for subjects submitted to total sleep deprivation and one-night of recovery.

**Methods:** Fourteen healthy men (26–37 years old) participated in an experimental cross-over design with two conditions: extended sleep (EXT, 9.8 ± 0.1 h of Time In Bed, TIB) and habitual sleep (HAB, 8.2 ± 0.1 h TIB). During these two conditions subjects underwent two consecutive phases: Six nights of either EXT or HAB followed by 3 days in-laboratory: baseline (BASE), TSD (38 h) and after recovery (REC). EFs capacities were assessed through Go-NoGo (inhibition) and 2N-Back (working memory) tasks. Both EFs capacities were measured at different time (BASE/TSD/REC: 09:30, 13:00, 16:00; TSD: 21:00, 00:00, 03:00, 06:30).

**Results:** In both conditions (HAB and EXT), TSD was associated with deficits in inhibition (higher errors and mean reaction time from TSD 09:30 until the end; *p* < 0.05) and working memory (lower corrects responses from TSD 06:30 or 09:30; *p* < 0.05). We observed no significant differences between HAB and EXT conditions on EFs capacities during BASE, TSD, and REC periods.

**Conclusion:** Six nights of sleep extension is neither efficient to reduce core EFs deficits related to TSD nor to improve such capacities after a recovery night. These results highlight that sleep extension (six nights of 10 h of TIB) is not effective to limit EFs deficits related to TSD suggesting a disconnection inside cognition between executive and sustained attention processes. Clinical Trials: NCT02352272.

## Statement of Significance

It is widely admitted that total sleep deprivation (TSD) is responsible for a large range of cognitive disturbances in healthy adults. With sleep extension it is possible to reduce alertness and sustained attention deficits related to TSD. The demonstration here of no beneficial effect of sleep extension on inhibition and working memory deficits-related to TSD would open the discussion upon a disconnection inside cognitive deficits related to TSD (attention versus executive processes).

## Introduction

Extreme lack of sleep (TSD) influences negatively many cognitive domains (sustained attention, executive, decision-making, and emotional processes) (Killgore, 2010; Krause et al., 2017). Core executive function (Core EF) is a broad term that encompasses some cognitive capacities making possible the functioning of higher-level EFs such as planning, problem solving and reasoning in order to achieve complex and goal-directed behavior (Diamond, 2013). Core EF includes inhibition (response inhibition and interference control), working memory (i.e., the transient monitoring, processing, and manipulation of task relevant information over a brief period of time) and cognitive flexibility (i.e., the ability to quickly and flexibly adapt to changing circumstances and to think “*outside the box*”) (Diamond, 2013).

In early 1970s, Taub et al. (1971) have shown that extending TIB (3 h of TIB, TIB during two nights) resulted in a significant higher TST (2 h). Later, Roehrs et al. (1989) have shown that a longer sleep extension period (10 h of TIB for six consecutive nights) was beneficial to objective sleepiness (Mean Sleep Latency Test) in healthy young subjects (21–35 years old) and more efficiently in sleepy subjects compared to alert ones (Roehrs et al., 1989). They confirmed, few years later, this hypothesis with 2 weeks of sleep extension (Roehrs et al., 1996). Subsequently, Mah et al. (2011) have shown that a 5–7 week sleep extension period was associated with a better athletic performance associated with a higher psychomotor vigilance in basket-players. However, this long sleep extension period raises the question about beneficial effect of sleep extension with shorter durations. More recently, it has been shown that 1 week of extended sleep (with 9.8 ± 0.1 h TIB) improves sustained attention and reduce sleep pressure at BASE (Rupp et al., 2009; Arnal et al., 2015). After 1 week of extended-sleep compared to habitual-sleep, subjects are lesser objectively sleepy (i.e., smaller latencies to fall asleep) and performed better in a PVT during acute prolonged wakefulness (38 h of TSD) (Arnal et al., 2015) and during a 7 days of sleep restriction (Rupp et al., 2009). Such findings have coined the term “*banking sleep*” to describe such extended sleep periods that improve performance in sleep restriction or TSD situations (Axelsson and Vyazovskiy, 2015).

It is well known that TSD is responsible for sustained attention deficits using PVT (Doran et al., 2001) for review see (Killgore, 2010) and is associated with a significant reduction of metabolic and neuronal activity in a parieto-frontal network for review see (Ma et al., 2015). Core EFs (such as behavioral inhibition, working memory, and cognitive flexibility) are essential to decisions in everyday life situations (i.e., planning, reasoning, problem solving, and decision making) (Miyake et al., 2000), for review see (Smith and Jonides, 1999; Diamond, 2013). They request optimal functioning of parieto-frontal networks and fronto-striatal networks, for review see (Nowrangi et al., 2014). Previous studies have pointed out cognitive deficits related to TSD such as core executive (Chee et al., 2006; Drummond et al., 2006; Tucker et al., 2010; Anderson and Platten, 2011) and decision-making ones [for review see (Killgore, 2010; Anderson and Platten, 2011; Krause et al., 2017)], suggesting a common pathway for cognitive deficits during TSD. However, others studies have highlighted a differential disruption of sustained attention and executive processes by TSD (Harrison et al., 2007; Lo et al., 2012) for review see (Jackson et al., 2013). For instance Schmidt et al. (2009) demonstrated a double influence of time awake (sleep pressure) and time of day (circadian) on sustained attention capacity and on the corresponding neural activity whereas Harrison et al. (2007) did not observe such circadian influences on executive capacity. More recently Lo and coworkers have demonstrated that working memory (assessed through a 1 to 3N-Back task), an executive function, is less affected by TSD than sustained attention and sleepiness (Lo et al., 2012). Overall, these findings challenge a dual (circadian and homeostatic) influence of sleep on all cognitive domains (sustained attention, executive, and decision-making processes) and raise the question of the potential effect of sleep extension on EFs (i.e., core EFs) during TSD. Sleep extension has been found to limit the degradation of sustained attention during TSD and the day following the first night of REC in healthy subjects (Lo et al., 2012).

The aim of this study was to test if there is a potential benefit of sleep extension (six nights) on two core EFs (inhibition and working memory) processes during TSD followed by one night of sleep REC in healthy subjects. We hypothesized that sleep extension would influence EFs capacities and at best limit the degradation during TSD and the day after the first REC night. But we cannot exclude the null hypothesis.

## Materials and Methods

### Subjects

Fourteen healthy right-handed men, aged 31.4 ± 3.9 years with a normal BMI (24.0 ± 2.0 Kg/m^2^), were included in this controlled study after receiving their informed written consent. The ethics committee of the Hotel Dieu – Ile de France 1 (Paris) and the French National Agency for the Safety of Medicines and Health Products (ANSM) approved the protocol (N°ID RCB: 2013-A01403-42). It was conducted according to the principles laid out in the Declaration of Helsinki of 1975, as revised in 2001. The subjects underwent a detailed medical history and examination including an electrocardiogram at rest. Exclusion criteria were: an excessive daytime sleepiness (Epworth Sleepiness Scales > 11) (Johns, 1991), bad sleep complaints (Pittsburg sleep quality index > 5) (Buysse et al., 1989), not being considered as an intermediate chronotype or moderately morning type on the Horne and Östberg questionnaire (<42 or >69) (Horne and Ostberg, 1976), scoring ≥ 13 on the Beck Depression Inventory (Beck et al., 1961), shift workers, daily smokers, daily consumers of alcohol and more than 400 mg of caffeine per day, a BMI > 28 Kg/m^2^, taking medication chronically (headache, allergy, etc.). All subjects that reported any of the following items during the previous month were excluded: (1) an average of night-sleep >9 h and <6 h from Sunday to Thursday, (2) a difference between week-nights and week-end night sleep > 45 min., (3) an average lights-out time earlier than 21:00 from Sunday through Thursday, (4) an average wake-up time later than 9:00 from Monday through Friday. Eighteen volunteers were initially selected and seen at a preliminary visit and four of them were not included, based on exclusion criteria.

To confirm these subjective data, actigraphy (Actiwatch TM, Cambridge Neurotechnology, Cambridgeshire, United Kingdom) was used during three consecutive weeks to objectively and accurately measure subject’s habitual sleep time during the nights of the week. Data were scored manually for TST (min), defined by the time of sleep within the identified sleep period (elapsed time from the start of sleep to sleep end time), for TIB and for awakening time. The reported differences between week and weekend nights were around 30 min and habitual TST during week nights were between 7.5 and 8 h per night.

### Protocol

**FIGURE 1:**
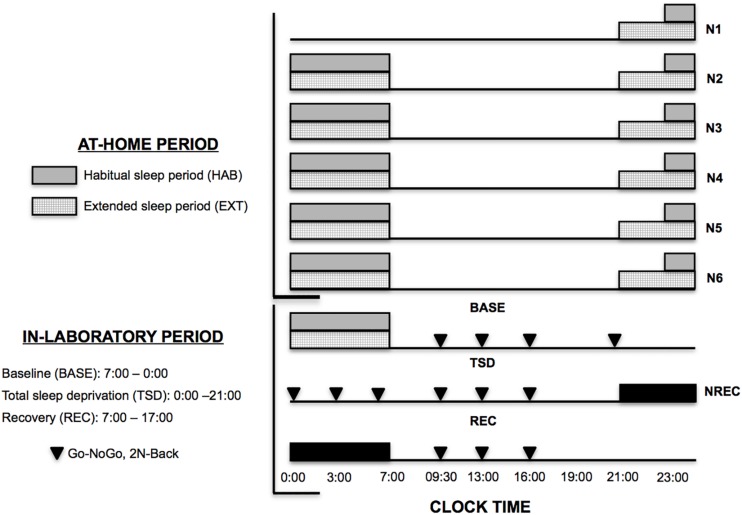
Experimental protocol. N: Night. NREC: Night Sleep Recovery. ▼, core executive tasks (Go-noGo and 2N-Back tasks). Sleep timing for habitual (HAB) (gray bars) and extended (EXT) sleep condition (hatched bars).

All subjects participated to an experimental randomized counter-balanced cross-over protocol with two sleep conditions: extended (EXT, 9.8 ± 0.1 h of TIB) and habitual night sleep (HAB, 8.2 ± 0.1 h TIB). The washout duration between these two experimental counter-balanced periods was 6 weeks. In each of these two conditions, subjects followed up two consecutive experimental periods: (1) Five nights of either EXT or HAB at home (N1 to N5, Figure 1) and then (2) 3 days in a sleep laboratory with a BASE night (N6, Figure 1), an extended wake period (TSD) and a REC night (NREC, Figure 1). 2 weeks before the first phase (At-Home period, Figure 1), a familiarization night was spent in the laboratory to avoid any first-night laboratory effect. Moreover, a control week with 8 h in bed was realized before first phase to avoid starting the experiment with the subjects in sleep debt. TIB and awakening time was checked during the control week with actigraphy.

During the first period (At-Home period, Figure 1), sleep nights were recorded by polysomnography (PSG) with miniaturized and wireless devices (Sauvet et al., 2014). In the HAB condition, subjects were instructed to maintain their habitual sleep and wake time and spend ≥8 h in bed (bedtime between 22:30 and wake up at 7:00). In the EXT condition, subjects spent 10 h of TIB bedtime at 21:00 and wake up time at 7:00. In these two experimental conditions wake up time was maintained at 7:00 am in order to accustom them to this wake-up time during the sleep-laboratory period (In-Laboratory period, Figure 1). The subjects were allowed to maintain their usual lifestyles but needed to return the PSG equipment to the laboratory every morning.

The second experimental period was conducted in a sleep laboratory and started at 17:00 after the 5th night at home (Figure 1). This 3 days in-laboratory period consisted with a BASE night (either EXT or HAB, N6), a BASE day (BASE: 07:00–00:00) followed by an extended wake period (TSD: 00:00–21:00), a REC night (NREC: 21:00–07:00), and a REC day (REC: 07:00–17:00) (Figure 1). During this entire experimental in-laboratory period, all subjects were PSG equipped with the same miniaturized and wireless devices (Sauvet et al., 2014) in order to continuously record their wake, sleep and micro-sleeps stages. The day of subject’s entry into this second experimental period was always the same day: Saturday.

### Testing Facilities During the Sleep-Laboratory Period

Subjects were all tested and sheltered individually in temperature-controlled (22 ± 1°C), 3 × 4 m rooms that contained a bed, restroom facilities and a computer workstation. Sleep laboratory illumination was maintained at 150–200 lux during this entire sleep-laboratory period with lights off during night sleep periods and that took place at the Hotel-Dieu APHP Hospital (Paris, France). Subjects were prohibited from exercise, caffeine, tobacco, alcohol or others psychoactive substances 48 h before and during the study. Meals and caloric intake were standardized for all subjects in accordance with the recommendations of the National Health Agency for Security Food, Environment and Labor (2600 kcal/day, providing 55% carbohydrate, 30% fat and 15% protein). For the majority of the population, this is the daily energy intake for men aged between 20 and 40 years under regular daily activities. Water was allowed without restriction. When not engaged in any specific testing or meals, subjects followed a standardized activity program (reading, watching TV or videos, playing video or card games). Six investigators were systematically present in the sleep-laboratory with at least two of them with the subjects. Two teams of 12-h shifts were organized to maintain a good level of investigator alertness. When the subjects were about to fall asleep (eyes closed, head down), they were gently and immediately woken up (i.e., no period of sleep 30 s).

### Night-Time Sleep Assessment

During N1 to N6 and NREC, an ultra-miniaturized wireless PSG device (Sauvet et al., 2014) was used to limit patient discomfort (Actiwave, CamNtech Ltd., Cambridge, United Kingdom) and provide continuous monitoring for 6 EEG (F3, C3, O1 and F4, C4, O2), 2 electrocardiograms and 2 electro-oculograms (outer canthus of each eye), and 2 electromyograms (chin). Contralateral mastoid leads served as references for all unipolar measurements (electroencephalograms and electro-oculograms). Two trained research technicians scored data of PSG, in accordance to AASM criteria (Silber et al., 2007), and using Somnologica software (TM, Medcare, Reykjavik, Iceland). Sleep period time, wakefulness after sleep onset, TST, sleep efficiency (TST/sleep period time), sleep onset latency (first epoch - 30 s of any sleep stage), and the time spend in various sleep stages (sleep stages 1, 2, 3, and REM) were determined. The PSG remained in place for further testing throughout the protocol.

For the following executive tasks, subjects performed tests at different time in this in-laboratory period (BASE: 9:30, 13:00, 16:00; TSD: 21:00, 00:00, 03:00, 07:00; 9:30, 13:00, 16:00; REC: 9:30, 13:00, 16:00; Figure 1). More detailed instructions and habituation to these two tests were provided during a familiarization day.

#### Go-NoGo Task: Executive Task N°1

In this first executive task, subject has to either respond or not respond when a stimulus arrived on a screen. After the appearance of a fixation cross in the center of the screen during 500 ms, an arrow appeared in the center of the screen during 1 s (Rabat et al., 2016). Depending on the test instruction, that changes in every session, subjects have to respond as quickly as possible when the arrow pointed out on the right (“*Go*” response) and not to respond when it pointed on the left (“*No-Go*” response). The proportion is always as follow: 67% of *“Go”* trials and 33% of *“No-Go”* trials (Rabat et al., 2016). Subjects have 2 s to respond and their response was directly followed by a new trial in order to determine the capacity of subjects to consciously inhibit non-relevant automated responses (motor inhibition process). The total duration of this task is around 7 min and 30 s. Time response, commission errors and omission were the three variables that are taken into account in this task (Rabat et al., 2016).

#### 2N-Back Working Memory Task: Executive Task N°2

In this second executive task [visual working memory task (Cohen et al., 1994)], pseudo random sequences of letters were presented to subjects and they have to respond to a pre-specified letter appearing on a computer screen (at the center). In this study, the visual working memory task included two conditions: a “working memory” 2-back condition (2-back: respond whenever the current letter is identical to the letter present two trials back, i.e., M-X-M) and a “control condition” 0-back condition (0-back: respond to a specific letter, i.e., W) that was related to a vigilance capacity. For this study, the working memory task contains three blocks of each condition (0-back and 2-back) that were pseudo randomly presented. Indeed, this task always started with a 0-back condition in order to help participants ease into the task. Inside each block that includes 24 trials, each trial is divided into three sequences: appearance of a fixation cross in the center of the screen (500 ms), appearance a consonant letter in the center of the screen (500 ms) and a fixation cross in the center of the screen (around 2500 ms) to allow subjects to either respond or not respond depending on the letter displayed on the computer screen. The total duration of this task is around 9 min. At the beginning of each block, brief instructions were displayed on the screen to inform subjects about whether the block was a 0 or a 2-back condition. More detailed instructions and habituation to the test were provided during a familiarization day. Time response and number of correct responses (both good responses and good non-responses) were the two variables that are taken into account in this task.

### Statistical Analysis

All data in text and figures are presented as mean ± standard error of the mean (SEM). Statistical analyses were performed using Statistica 10.0 (StatSoft^®^). All value’s distributions were tested for their normality (Kolmogorov–Smirnov, Shapiro–Wilk and Liliefors tests). When the value’s distribution was normal (almost two positive tests), a two-way repeated-measures ANOVAs were conducted on sleep parameters (condition × night) from N1 to NREC, on Go-NoGo and 2N-Back parameters (time since awakening × condition) in order to evaluate timeline effect, condition and interaction between these two parameters. When ANOVA results revealed significant main effect (time since awakening/condition) and/or interactions, we used Newman-Keuls (NK) or Duncan (D) *post hoc* tests to identify differences inside (timeline effect) and between EXT and HAB conditions in BASE, TSD and REC. When time since awakening effect was revealed, all points were compared to a single control value (09:30 at BASE).

When value’s distribution was not normal, we used a one way non-parametric ANOVA test with repeated measures (ANOVA of Friedman and Wilcoxon test for *post hoc* comparison: W) to identify differences inside (timeline effect) each condition (EXT and HAB) in BASE, TSD and REC. Non-parametric *T*-tests (Mann–Whitney *U*-test: MW) were used to identify differences at a time point (i.e., 09:30 at TSD) between each condition EXT and HAB.

Statistical significance was set at *p* < 0.05 for all statistical analyses.

## Results

### Night-Time Sleep Assessment

There was no difference (*p* = 0.97) for TIB and awakening time between habitual (HAB; 8.0 ± 0.3 h) and extended (EXT; 8.0 ± 0.2 h) conditions during the control week. Over the six nights of sleep extension, we observed a significant condition effect for TIB [*F*(1,12) = 151.5; *p* < 10^–3^], for TST [*F*(1,12) = 164.8; *p* < 10^–3^] and for WASO [*F*(1,12) = 5.9; *p* < 0.05]. We neither observed significant condition effect for sleep latency [*F*(1,12) = 0.1; *p* > 0.10] and sleep efficiency [*F*(1,12) = 0.1; *p* > 0.10]. Over the six nights, subjects spent each night an average TIB and TST of 8.2 ± 0.1 and 7.0 ± 0.1 h for HAB condition and 9.8 ± 0.1 and 8.2 ± 0.1 h for EXT condition, respectively (significant condition main effect for both). Three of the sleep stages duration were significantly higher in EXT compared to HAB condition [stage N1: *F*(1,12) = 34.9; *p* < 10^–3^, stage N2: *F*(1,12) = 19.4; *p* < 10^–3^, and REM sleep stage: *F*(1,12) = 10.2; *p* < 10^–3^], while stage N3 was not different [*F*(1,12) = 17.2; *p* > 0.10]. As mentioned in Table 1, sleep period time, TST and sleep duration for stage 1, 2, and REM were significantly higher during the last night in sleep extension condition (N6-EXT) compared to the last night in habitual condition (N6-HAB) without any significant increase of the duration for stage N3. Furthermore, the night of sleep recovery (NREC) is associated with a significant decrease of sleep latency, of WASO and of the duration of stage N1 and with a significant increase of stage N3 for both conditions (HAB and EXT, Table 1).

**TABLE 1:** Mean ± SD for sleep parameters during the night before total sleep deprivation (N6) and the night of sleep recovery (NREC) in habitual (HAB) and extended (EXT) conditions.

		**N6**	**NREC**	
	**HAB**	**EXT**	**HAB**	**EXT**
**Sleep continuity**				
Sleep Period Time (min)	458±3	$576\pm 6^{\#\,\#\,\#}$	$570\pm 5^{*,**}$	568±4
Total Sleep Time (min)	411±8	$513\pm 10^{\#\,\#\,\#}$	$543\pm 6^{*,**}$	545±3
Sleep efficiency (%)	85±2	85±2	$94\pm 1^{*,**}$	$95\pm 1^{*,**}$
Sleep latency (min)	25±4	25±4	$6\pm 1^{*,**}$	$6\pm 2^{*,**}$
WASO (min)	47±6	63±8	$27\pm 6^{*}$	$24\pm 4^{*}$
**Sleep duration**				
Stage N1 (min)	20±2	$34\pm 4^{\#\,\#\,\#}$	$7\pm 1^{*,**}$	$9\pm 2^{*,**}$
Stage N2 (min)	175±9	$222\pm 11^{\#\,\#\,\#}$	193±9	195±11
Stage N3 (min)	132±7	149±12	$231\pm 13^{*,**}$	$222\pm 10^{*,**}$
Stage REM (min)	84±6	$109\pm 7^{\#\,\#\,\#}$	$112\pm 9^{*}$	119±4

EXT versus HAB: ^###^*p* < 0.001. N6 versus NREC: ^*^p < 0.05 and ^∗∗∗^*p* < 0.001. WASO, wake after sleep onset; REM, rapid eye movement sleep.

### Go-NoGo Task

**FIGURE 2:**
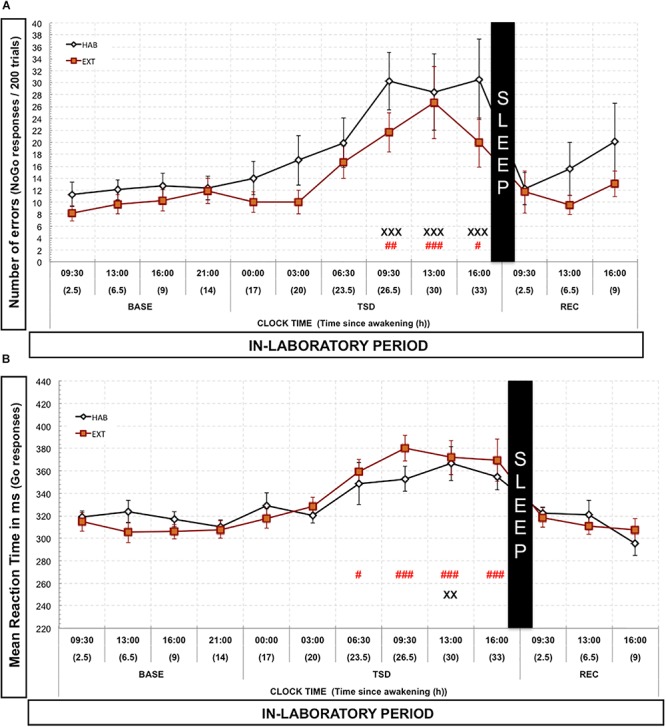
Number of errors **(A)** and mean reaction time of correct responses **(B)** in the Go-noGo task. Number of commission errors (“No-Go” responses, black or red lines) is represented as the mean (±SEM) for 14 subjects during baseline (BASE), total sleep deprivation (TSD) and recovery (REC) periods. X: Significantly different from 09:30 BASE value for the habitual (HAB) sleep condition (XX for *p* < 0.01; and XXX for *p* < 0.001). # Significantly different from 09:30 BASE value for the extended (EXT) condition (# for *p* < 0.05; ## for *p* < 0.01; and ### for *p* < 0.001).

Concerning the number of errors (errors due to no-go responses), we observed a significant time since awakening effect [*F*(1,12) = 11.20; *p* < 10^–6^] but with no condition effect [*F*(1,1) = 1.44; *p* > 0.24] and with no time since awakening × condition interaction effect [*F*(1,12) = 0.748; *p* > 0.74]. Compared to 09:30 BASE value, the number of errors significantly increased during TSD, from 09:30 to 16:00, both for HAB and EXT conditions (Figure 2A). Regarding reaction time of correct responses, there was a significant time since awakening effect [*F*(1,12) = 16.145; *p* < 10^–6^] but with no condition effect [*F*(1,1) = 0.019; *p* > 0.89] and with no time since awakening × condition interaction effect [*F*(1,12) = 1.188; *p* > 0.29]. Compared to 09:30 BASE value, reaction time of correct responses significantly increased during TSD, respectively, at 13:30 and from 06:30 to 16:00 for HAB and EXT conditions (Figure 2B). We only found one significant and negative correlation between variation of N2 sleep during NREC and variation of the mean reaction time over the REC day (variation = EXT minus HAB) (*r* = −0.646; *p* < 0.05).

### 2N-Back Task

**FIGURE 3:**
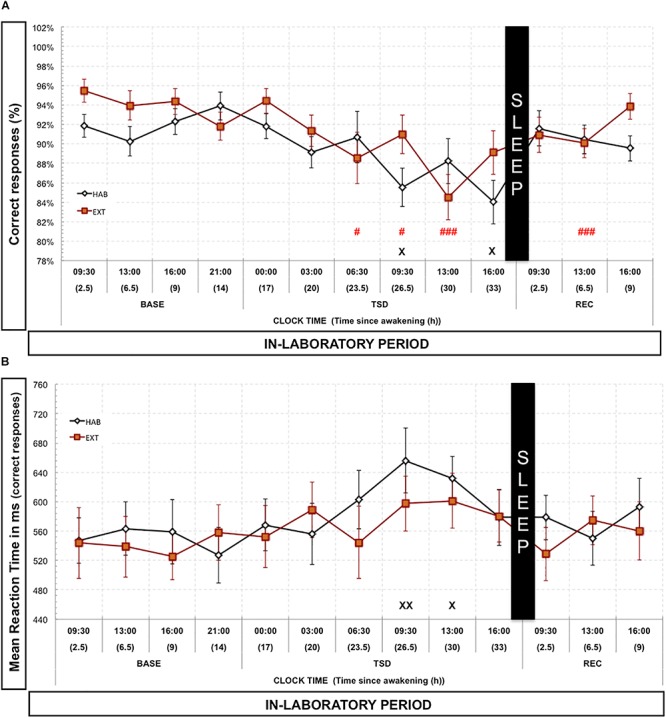
Percentage of correct responses **(A)** and mean reaction time **(B)** in the 2N-Back task. Percentage of correct responses (black and red lines) is represented as the mean (±SEM) for 14 subjects during baseline (BASE), total sleep deprivation (TSD) and recovery (REC) periods. X: Significantly different from 09:30 BASE value for the habitual (HAB) sleep condition (X for *p* < 0.05; XX for *p* < 0.01). # Significantly different from 09:30 BASE value for the extended (EXT) condition (# for *p* < 0.05; and ### for *p* < 0.001).

Regarding the 2N-Back condition, we observed a significant time since awakening effect for the proportion of correct responses in both HAB [Chi^2^ (14,12) = 25.35; *p* < 0.013] and EXT conditions [Chi^2^ (14,12) = 31.76; *p* < 0.0015]. We did not observe any significant differences between HAB and EXT conditions for the different timeline points (MW: *p* > 0.14; *p* > 0.35; *p* > 0.19; *p* > 0.35; *p* > 0.60; *p* > 0.76; *p* > 0.94; *p* > 0.11; *p* > 0.24; *p* > 0.13; *p* > 0.80; *p* > 0.51; *p* > 0.37, respectively, for 09:30, 13:00, 16:00, and 21:00 in BASE; 00:00, 03:00, 06:30, 09:30, 13:00, and 16:00 in TSD and 09:30, 13:00, and 16:00 in REC). Compared to 09:30 BASE value, the proportion of correct responses decreased significantly during TSD at 09:30 and 16:00 in HAB and from 06:30 to 13:00 in EXT conditions and also at 13:00 in REC for the EXT condition (Figure 3A). Concerning reaction time of correct responses, there was a significant time since awakening effect [*F*(1,12) = 2.41; *p* < 0.0053] but with no condition effect [*F*(1,1) = 0.148; *p* > 0.70] and no time since awakening × condition interaction effect [*F*(1,12) = 0.872; *p* > 0.57]. Compared to 09:30 BASE value, reaction time of correct responses significantly increased during TSD at 09:30 and 13:30 but only for HAB condition (Figure 3B). We only found one significant and positive correlation between variation of N2 sleep during N6 and the mean variation of the percentage of correct responses over the sleep deprivation day (variation = EXT minus HAB) (*r* = 0.575; *p* < 0.05). The variation of REM sleep during NREC was also positively correlated with the mean variation of the percentage of correct responses over the REC day (variation = EXT minus HAB) (*r* = 0.570; *p* < 0.05).

**FIGURE 4:**
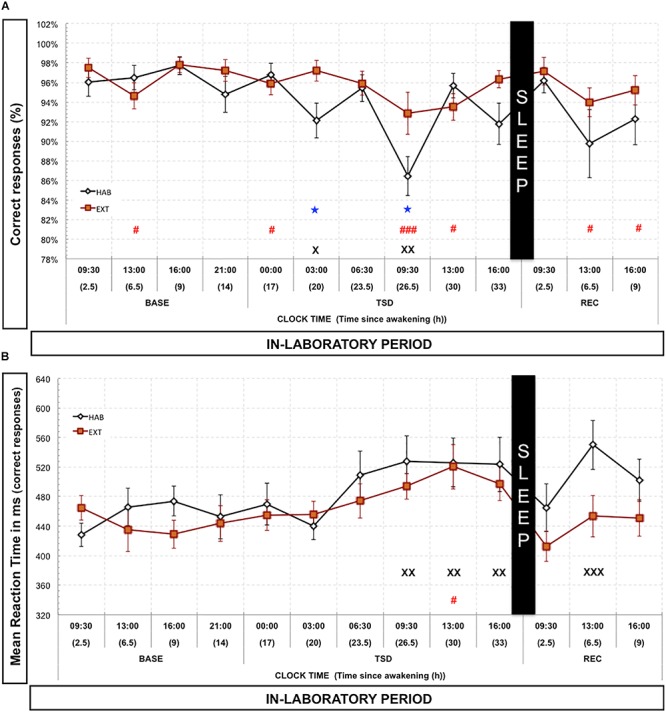
Percentage of correct responses **(A)** and mean reaction time **(B)** in the 0N-Back task (vigilance control condition). Percentage of correct responses (black and red lines) is represented as the mean (±SEM) for 14 subjects during baseline (BASE), total sleep deprivation (TSD), and recovery (REC) periods. X: Significantly different from 09:30 BASE value for the habitual (HAB) sleep condition (X for *p* < 0.05; XX for *p* < 0.01; and XXX for *p* < 0.001). # Significantly different from 9:30 BASE value for the extended (EXT) condition (# for p < 0.05; and ### for *p* < 0.001). ★ Significantly different between habitual (HAB) and extended (EXT) conditions (*p* < 0.05).

Regarding the 0N-Back condition (control-vigilance condition), we observed a significant time since awakening effect for the proportion of correct responses in both HAB [Chi^2^ (14,12) = 32.75; *p* < 0.001] and EXT conditions [Chi^2^ (14,12) = 26.61; *p* < 0.017]. The proportion of correct responses significantly decreased during TSD at 03:00 and 09:30 compared to 09:30 BASE value for HAB, and also decreased during BASE (13:00), during TSD (00:00, 09:30, and 13:00) and during REC (13:00 and 16:00) compared to 09:30 BASE value for EXT condition (Figure 4A). We observed significant differences between HAB and EXT conditions for two timeline points during TSD (MW: *p* < 0.05 for 03:00 and 09:30, Figure 4A). Concerning reaction time of correct responses, there was a significant time since awakening effect [*F*(1,12) = 5.52; *p* < 10^–6^] but with no condition effect [*F*(1,1) = 0.914; *p* > 0.34] and no time since awakening × condition interaction effect [*F*(1,12) = 1.70; *p* > 0.06]. Compared to 09:30 BASE value, reaction time of correct responses significantly increased during TSD from 09:30 to 16:00 and at 13:00 during REC in HAB condition and only during TSD at 13:00 in EXT condition (Figure 4B).

## Discussion

The main finding of this randomized crossover study is that sleep extension is not efficient to reduce two core EFs deficits (i.e., inhibition and working memory) induced by a 38-h TSD protocol in our group of healthy male subjects. The inhibition capacity was assessed through the Go-NoGo task, which evaluates the ability to withhold a motor response, and working memory through a digit N-back paradigm with a 2N-back load as a working memory condition and a 0N-back load as a vigilance control condition. Our results only showed a higher percentage of correct responses in the sleep extension condition compared to the habitual sleep one at two time points during TSD (in the night and early morning). At last, our results pointed out an increase of TST of 72 min associated with significant increases of N1, N2, and REM but not N3 sleep amounts at the sixth night of sleep extension.

In our study, we cannot exclude for a beneficial effect of sleep extension on core EFs deficits during TSD but such banking sleep will have to be stronger than the one we used here (either for the number of nights > 6 nights and for the nightly increase of TST > 72 min). Interestingly, Arnal et al. previously demonstrated that six nights of sleep extension were sufficient to sustain more efficiently the awake (less involuntary micro sleeps) and sustained attention (less PVT Lapses) capacities before and during a TSD period (Arnal et al., 2015). Our results are both in accordance with Drummond et al. (2006, 2012) reports suggesting differential disturbances of sustained attention and EFs processes under TSD and with Lo et al. (2012) findings showing a small effect size of 1 week of sleep history (control or sleep restriction) on working memory deficits related to TSD compared to a higher one for sleepiness and sustained attention processes. More recently, Slama et al. (2017) have shown differentiated effects of one night of TSD on sustained attention and EFs (flexibility, inhibition, and working memory) with deterioration in attention, flexibility, and inhibition capacities and not for working memory one (Slama et al., 2017). Besides de Bruin et al. (2017) concluded, in a recently review, to an indirect detrimental effect of TSD on working memory of adolescents due to other cognitive sub-domains deficits such as attention. In support of that, our results highlight a beneficial effect of banking sleep on the 0N-Back condition (control) in the working memory task (i.e., correct responses only) at two key times for alertness (3:00 and 9:30 a.m. during TSD). In an interesting way, this 0N-back condition controls the capacity of subjects to detect a target letter among many others letters and thus account for a sustained attention-like capacity, known to be highly disturbed by a lack of sleep (both with TSD and sleep restriction) (Graw et al., 2004; Maire et al., 2014). This result confirms the previous finding on beneficial effects of banking sleep on sustained attention process (Arnal et al., 2015).

Our results also point out a differential kinetic of the two core EFs deficits during TSD (i.e., from 26.5 h) compared to sustained attention ones (at a later time 20 h) (Arnal et al., 2015). This finding agrees with previous observations showing that working memory capacity seems to be less rapidly affected by TSD (41 h of prolonged wakefulness) than sleepiness and sustained attention process (Lo et al., 2012; Slama et al., 2017). In agreement with Frenda and Fenn (2016), we can also suggest that TSD impairs cognitive processes by primarily inhibiting the ability of individuals to be alert and to sustain their attention, leaving their other cognitive functions under compensatory brain responses. This hypothesis relied on studies showing a better working memory performance at most difficult load after 35 h of TSD which was related to increased cerebral responses within parieto-frontal networks (Drummond et al., 2004, 2005b, 2012; Lythe et al., 2012).

The attention and inhibition processes engage activation of different frontal brain regions with an activation of the right dorsolateral prefrontal cortex for sustained attention (Culham et al., 2001; Yamasaki et al., 2002) and right ventro-lateral part for inhibition (Aron et al., 2004). Since prolonged wakefulness is associated with significant and specific reductions of the metabolism of fronto-parietal networks (Wu et al., 1991; Thomas et al., 2000; Ma et al., 2015), notably those dedicated to sustained attention (Drummond et al., 2005a), we hypothesize that banking sleep, beneficial to sustained attention (Arnal et al., 2015), would help metabolism of such brain networks and not to the entire whole brain networks (i.e., ventral part of the prefrontal cortex). In their meta-analyses review of neuroimaging studies regarding attention deficits under TSD, Ma et al. (2015) pointed out that TSD increases thalamic activation that would reflect a complex interaction between sleep loss (de-arousing effects) and task engagement (arousal effect). This could be supported by studies of Drummond et al. (2004, 2005b) showing increased cerebral responses (i.e., left inferior frontal gyrus, dorsolateral prefrontal cortex, bilateral inferior parietal lobe, and bilateral temporal cortex) with task difficulty in subjects engaged either in a verbal learning or in logical reasoning task during 35–36 h of TSD leaving their performance level not affected.

In our study, absence of beneficial effect of sleep extension on EFs while it was found on sustained attention (Roehrs et al., 1989) suggest that core EFs would be indirectly connected (through attention) to the wake capacity while sustained attention would be directly connected. This hypothesis is in agreement with previous studies showing that sustained attention is a cognitive domain that is robustly affected by sleep loss in contrary to EFs (Lo et al., 2012; Van Someren et al., 2015; de Bruin et al., 2017). Besides, no circadian effect for executive processes in a TSD protocol has been revealed (Harrison et al., 2007; Bratzke et al., 2012; Sagaspe et al., 2012) whereas a time of day variation in sustained attention capacity in large sample of adult subjects (6,363 subjects) (Riley et al., 2017) is in agreement with our hypothesis that sustained attention (and maybe attention in general) is strongly both influenced, as alertness, by two main factors responsible for the sleep-wake regulation (Dijk and von Schantz, 2005; Brown et al., 2012). To a larger extent, studies showing either a morning time window of vulnerability for sustained attention processes (Cohen et al., 2010; Mollicone et al., 2010; Lo et al., 2012) or a differential kinetic between sustained attention and EFs (i.e., inhibition) in chronic sleep restricted subjects (Rabat et al., 2016) are also in accordance with this hypothesis. Intriguingly recently, Frenda and Fenn (2016) pointed out that pharmacological stimulants (i.e., caffeine, modafinil, and dextroamphetamine), logically awakening substances, are all efficient to compensate for sustained attention deficits related to TSD but not so for EFs (Wesensten et al., 2002, 2005; Killgore et al., 2009) [for review see (Frenda and Fenn, 2016)].

The fact that sleep extension, beneficial for awake and sustained attention capacities, is not associated with a significant increase of SWS (N3 sleep) (Rajaratnam et al., 2004; Rupp et al., 2009; Arnal et al., 2015), raised the question of the clear functional role of deep sleep in the wake and behavioral capacity of subjects submitted to acute (TSD) or chronic (CSR) sleep debt (Leger et al., 2018). This is strengthened by absence of significant correlation between variation (EXT minus HAB) of N3 sleep in the night preceding sleep deprivation (N6) or in the recovery night (NREC) and variation of EFs parameters over the sleep deprivation day and the sleep REC day, respectively. However, the significant and positive correlation that was found between variation of N2 sleep amount during the night preceding sleep deprivation (N6) and variation of the percentage of correct responses in the 2N-Back task (working memory) over the day of sleep deprivation is an indirect argument in favor of the significant contribution of sleep (quantity and quality) in a better stability and quality of wakefulness affected during TSD (Doran et al., 2001; Rupp et al., 2009; Arnal et al., 2015; Axelsson and Vyazovskiy, 2015). Since sleep extension is efficient to reduce micro-sleep and sustained attention deficits related to TSD (Arnal et al., 2015) and to down-regulate adenosine receptors (A1) levels in the frontal cortex and not the hippocampus (Chennaoui et al., 2017), our results could confirm that banking sleep would efficiency but indirectly promote wakefulness through modifications of neurotransmissions in key part of the brain implicated in sleep-wake regulation.

Regarding sleep extension effects on inhibition capacity (i.e., the Go-NoGo task) during TSD, our results showed a slight trend toward less errors in the extended sleep condition compared to the habitual one which suggested that the sample size is still fairly modest even though the cross-over design substantially increase its power. It would be interesting to investigate sleep extension effects on alertness, sustained attention and executive processes capacities during TSD in a larger group of healthy individuals and/or longer periods of banking sleep. There is also interest in evaluating sleep extension in female participants since a recent study showed a stronger nighttime impairment in cognitive performances in women than in men (Santhi et al., 2016).

To our knowledge this is the first study pointing out that sleep extension is neither efficient to erase nor to restrict EFs deficits related to TSD in healthy male subjects, while it was previously found beneficial for alertness and sustained attention deficits (Arnal et al., 2015). This study is in accordance with previous hypothesis stressing that sustained attention processes are more connected and influenced by alertness whereas core EFs less (Rabat et al., 2016). In previous studies using banking sleep in TSD or in chronic sleep restriction paradigm (Rupp et al., 2009; Arnal et al., 2015), beneficial effects for awake and sustained attention capacities were not associated with a significant increase of NREM (N3) sleep. As banking sleep did not improve EFs in our study, this raised the question on the functional role of N3 sleep on behavioral capacities of sleep-deprived individuals (Leger et al., 2018). From a practical point of view our work showing no efficiency of sleep extension on EFs deficits related to TSD, emphasizes that the management of cognition in various professional fields impacted by sleep debt (military, shift workers, long-distance drivers, health workers, etc.) has to take into account multidimensional aspects of cognition. It would also be important to study other non-pharmacological countermeasures to deal with this kind of problem.

## Ethics Statement

Clinical Trials Number: NCT02352272. The ethics committee of the Hotel Dieu – Ile de France 1 (Paris) and the French National Agency for the Safety of Medicines and Health Products (ANSM) approved the protocol (N°ID RCB: 2013-A01403-42).

## Author Contributions

AR, PA, HM, PVB, CB, CD, MG, FS, DL, DG-M, and MC conceived and designed the study. AR, PA, HM, ME, PVB, CB, CD, MG, and FS acquired the data. AR, HM, MG, and FS analyzed the data. AR, FS, DG-M, and MC interpreted the data and wrote the manuscript.

## Disclaimer

This research was not an industry-supported study. This work was carried out in the French Armed Forces Biomedical Research Institute (IRBA) located at Brétigny-sur-Orge, France. The Company PSA Peugeot Citroen now employs Clément Bougard as a Research Engineer in Cognitive Sciences. But, this company did not employ him at the time of this study. The same declaration is valid for Pierrick J. Arnal and the Dreem Company.

## Conflict of Interest Statement

The authors declare that the research was conducted in the absence of any commercial or financial relationships that could be construed as a potential conflict of interest.

## Abbreviations

BASE: baseline
BMI: body mass index
EFs: executive functions
EXT: extended night sleep group
HAB: habitual night sleep group
KSS: karolinska sleepiness scale
NREM Sleep: non-rapid eye movement sleep
PSQI: Pittsburgh Sleep Quality Index
PVT: psychomotor vigilance task
REC: recovery
SWS: slow wave sleep
TIB: time in bed
TSD: total sleep deprivation
TST: total sleep time.
